# A Cadaveric Case Report of an Anomalous Sural Nerve Course Through the Gastrocnemius

**DOI:** 10.7759/cureus.108348

**Published:** 2026-05-06

**Authors:** Iannis K Moshovitis, Naman Sahota, Palak Dutta

**Affiliations:** 1 Cardiology/General Surgery, William Carey University College of Osteopathic Medicine, Hattiesburg, USA; 2 Medicine, William Carey University College of Osteopathic Medicine, Hattiesburg, USA; 3 Surgery, University of Illinois, Chicago, USA

**Keywords:** anatomical variation, developmental anomaly, gastrocnemius penetration, lower extremity, neurovascular dissection, posterior leg surgery, sural nerve pathway

## Abstract

The sural nerve is a superficial sensory nerve of the posterior leg that innervates the lateral foot. Anatomical variations in its course are clinically relevant, as they may affect surgical procedures, nerve blocks, and the risk of iatrogenic injury. This case report describes a rare variant in which the sural nerve pierces between the two heads of the gastrocnemius muscle, a course infrequently reported in the literature and important for surgical awareness. During routine lower-extremity dissection by first-year medical students at William Carey College of Osteopathic Medicine, Hattiesburg, USA, a rare sural nerve variant was identified in a 72-year-old Caucasian female cadaver obtained from the University of South Alabama Anatomical Gift Program, with dementia listed as the cause of death. The right sural nerve pierced both the medial and lateral heads of the gastrocnemius muscle approximately 11.43 cm inferior to the center of the popliteal fossa, deviating from its typical superficial course. After traversing the muscle, the nerve continued normally along the posterior leg. Adjacent structures, including the popliteal artery, popliteal vein, and tibial nerve, were unremarkable. Intact muscle fibers and the absence of fibrosis support this finding as a benign anatomical variation. While sural nerve variations are well-documented, instances of the nerve piercing the gastrocnemius muscle are exceedingly rare. Damage to the sural nerve can result in sensory deficits affecting the lateral foot and lower leg, which may impair recovery and quality of life. Awareness of such variations is vital for clinicians to prevent inadvertent nerve injury during surgeries or nerve blocks.

## Introduction

The sural nerve is a sensory nerve that typically innervates the posterolateral aspect of the distal leg and the lateral foot. Its anatomical course and formation are highly variable, but it most commonly arises from the union of the medial sural cutaneous nerve (MSCN, branch of the tibial nerve) and the lateral sural cutaneous nerve (LSCN, branch of the common fibular nerve). This union most frequently occurs in the lower third of the leg [1,2].

With respect to the gastrocnemius muscle, the sural nerve typically descends superficially along the posterior leg, most often coursing between the medial and lateral heads without penetrating them. Rare anatomical variants, however, have been reported in which the sural nerve traverses directly through both heads of the gastrocnemius [3-5]. This variant is notable, with one cadaveric study identifying only a single case among 24 dissected legs [4]. Recognition of this anomaly carries clinical importance, as it may increase the risk of iatrogenic sural nerve injury during procedures involving the gastrocnemius, including muscle biopsies, tendon repairs, and nerve graft harvesting [4].

Awareness of this rare anatomical variant is essential for surgeons and clinicians to reduce the risk of iatrogenic nerve injury and optimize surgical outcomes. The considerable variability in sural nerve anatomy highlights the importance of meticulous dissection and thorough preoperative planning in procedures involving the posterior leg.

## Case presentation

A rare sural nerve pathway was identified during routine anatomical dissection of the right lower extremity in a 72-year-old Caucasian female cadaver donated through the University of Southern Alabama Anatomical Gift Program. The donor's cause of death was listed as dementia.

Dissection followed the protocols outlined in Grant's Dissector, 16th Edition, with the objective of evaluating the course of the sural nerve and its relationship to the gastrocnemius muscle while preserving surrounding structures [5]. Initially, the cadaver was positioned supine, and a longitudinal incision was made from the anterior superior iliac spine, extending medially across the thigh, over the patella, and to the dorsum of the foot, with transverse incisions added to facilitate skin removal while preserving superficial structures [1]. The cadaver was then repositioned prone, and posterior midline incisions were made from the gluteal fold to the heel to expose the fascia and neurovascular structures of the posterior lower limb [5].

The superficial fascia was examined, and the small saphenous vein and sural nerve were identified and cleaned. The sural nerve was observed coursing along the posterior leg, paralleling the small saphenous vein and piercing the deep fascia approximately midway down the calf [5]. Blunt dissection was used to trace the nerve from its origin to its termination near the lateral foot, with additional cutaneous nerves, including the posterior cutaneous nerve of the thigh, also identified.

**Figure 1:**
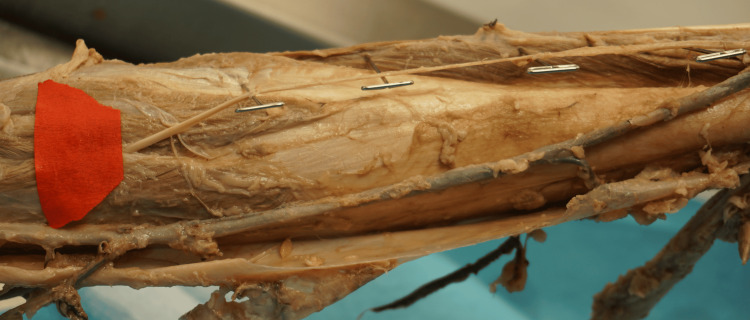
The red marker illustrates the unique anatomical variation of the sural nerve as it pierces through the lateral and medial heads of the gastrocnemius muscle. The precise location of this penetration is measured at 4.5 inches (11.43 centimeters) inferior to the center of the popliteal fossa.

**Figure 2:**
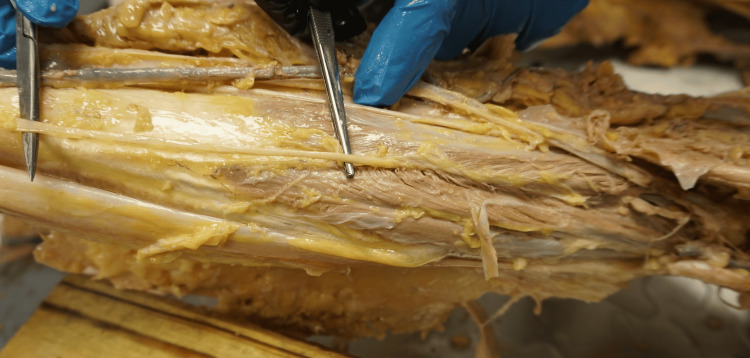
The forceps highlight the point where the sural nerve penetrates the lateral and medial heads of the gastrocnemius muscle, offering a clear view of this unique anatomical variation. This close-up image provides a detailed perspective, emphasizing the nerve path as it passes through the muscle tissue.

An unusual anatomical variation was noted in which the sural nerve pierced both the lateral and medial heads of the gastrocnemius approximately 4.5 inches (11.4 cm) inferior to the center of the popliteal fossa (see Figure 1). The nerve traversed the muscle bellies before resuming its typical distal course along the posterior leg (refer to Figure 2), representing a clear deviation from its usual superficial pathway.

The surrounding muscle fibers appeared undisturbed, suggesting a developmental adaptation rather than an acquired change. No abnormalities were observed in adjacent structures, including the popliteal artery, popliteal vein, or tibial nerve. Additionally, the absence of fibrosis or other pathological findings supports that this variation represents a benign developmental anomaly.

## Discussion

Anatomical variations of the sural nerve are well documented, reflecting its developmental complexity and diverse patterns of formation. In most individuals, the sural nerve arises from the convergence of the MSCN and LSCN, typically in the distal third of the leg [2]. However, both its origin and course demonstrate significant variability [6]. Despite this, penetration of the sural nerve through the gastrocnemius muscle remains exceedingly rare. Aktan Ikiz et al. [6] described cases in which the nerve coursed deep to or within the gastrocnemius heads, while Jeon et al. [7] identified only one such instance among 24 lower limbs. These findings highlight that intramuscular penetration is an uncommon but recognized variant.

Although often clinically silent, sural nerve variations may have important implications. The nerve is frequently utilized in grafting procedures, evaluated in nerve conduction studies, and encountered in posterior leg surgeries [8,9]. Variability in its depth or pathway, particularly intramuscular courses, may increase the risk of iatrogenic injury. Aberrant pathways have been associated with inadvertent transection during gastrocnemius tendon procedures, fasciotomies, and muscle biopsies [8]. Additionally, altered anatomy may predispose the nerve to compressive neuropathies, which, though uncommon, have been reported in the setting of trauma, fascial thickening, and mass lesions [10]. Patients typically present with sensory disturbances along the lateral foot and posterolateral calf, including burning pain, numbness, or paresthesia [10].

In our case, the sural nerve penetrated both the lateral and medial heads of the gastrocnemius approximately 4.5 inches inferior to the popliteal fossa, representing a marked deviation from its typical superficial course. The surrounding muscle fibers appeared undisturbed, suggesting a developmental rather than post-traumatic origin. This configuration may result from variations in limb bud growth, nerve patterning, or connective tissue development during embryogenesis [10], with the gastrocnemius potentially forming around the nerve. Similar to vascular compression phenomena such as trigeminal neuralgia from contact with the superior cerebellar artery [9], this intramuscular course places the nerve within a dynamic environment where mechanical irritation is possible. Although no pathological changes were observed in this cadaveric specimen, repetitive contraction of the gastrocnemius could theoretically increase intramuscular pressure and produce exertional neuropathic symptoms in vivo.

This case is further distinguished by the nerve passing cleanly through both heads of the gastrocnemius without associated structural abnormalities. Unlike prior reports involving accessory muscle slips or fascial entrapment [6-8], the surrounding anatomy was otherwise normal. However, this variation may complicate sural nerve harvesting and increase the risk of injury during procedures such as tendon repair, Strayer procedures, and fasciotomies. It may also affect nerve conduction studies, as intramuscular segments can be more difficult to stimulate and may require modified electrode placement [6].

Overall, this case highlights the plasticity of peripheral nerve development and the clinical importance of recognizing rare anatomical deviations. Similar to intramuscular courses of the musculocutaneous nerve [7] and variant tibial nerve bifurcations within the tarsal tunnel [6], this finding reinforces that peripheral nerves may adopt unconventional trajectories with meaningful surgical and diagnostic implications.

## Conclusions

This case report documents a rare instance of sural nerve penetration of the gastrocnemius muscle. Recognizing such anomalies is essential for clinicians, surgeons, and anatomists, as an unexpected nerve pathway may increase the risk of iatrogenic injuries during lower limb surgeries, complicate nerve block procedures, or influence nerve grafting outcomes. By documenting and disseminating findings of this variation, we contribute to the growing body of literature aimed at improving surgical planning, enhancing patient safety, and guiding future clinical and anatomical research.
